# Cover Crop Root Exudates Impact Soil Microbiome Functional Trajectories in Agricultural Soils

**DOI:** 10.21203/rs.3.rs-3956430/v1

**Published:** 2024-02-16

**Authors:** Valerie A. Seitz, Bridget B. McGivem, Mikayla A. Borton, Jacqueline M. Chaparro, Meagan E. Schipanski, Jessica E. Prenni, Kelly C. Wrighton

**Affiliations:** Colorado State University; Colorado State University; Colorado State University; Colorado State University; Colorado State University; Colorado State University; Colorado State University

**Keywords:** Phytohormone, plant growth promoting, metagenome assembled genome (MAG), liquid chromatography mass spectrometry (LC-MS), metatranscriptomics, Sorghum bicolor, Vicia villosa, Brassica napus, Secale cereal

## Abstract

**Background:**

Cover cropping is an agricultural practice that uses secondary crops to support the growth of primary crops through various mechanisms including erosion control, weed suppression, nutrient management, and enhanced biodiversity. Cover crops may elicit some of these ecosystem services through chemical interactions with the soil microbiome via root exudation, or the release of plant metabolites from roots. Phytohormones are one metabolite type exuded by plants that activate the rhizosphere microbiome, yet managing this chemical interaction remains an untapped mechanism for optimizing plant-soil microbiome interactions. Currently, there is limited understanding on the diversity of cover crop phytohormone root exudation patterns and how these chemical messages selectively enrich specific microbial taxa and functionalities in agricultural soils.

**Results:**

Here, we link variability in cover crop root exudate composition to changes in soil microbiome functionality. Exudate chemical profiles from 4 cover crop species (*Sorghum bicolor, Vicia villosa, Brassica napus,* and *Secale cereal*) were used as the chemical inputs to decipher microbial responses. These distinct exudate profiles, along with a no exudate control, were amended to agricultural soil microcosms with microbial responses tracked over time using metabolomes and genome-resolved metatranscriptomes. Our findings illustrated microbial metabolic patterns were unique in response to cover crop exudate inputs over time, particularly by sorghum and cereal rye amended microcosms where we identify novel microbial members (at the genera and family level) who produced IAA and GA_4_ over time. We also identify broad changes in microbial nitrogen cycling in response chemical inputs.

**Conclusions:**

We highlight that root exudate amendments alter microbial community function and phytohormone metabolisms, particularly in response to root exudates isolated from cereal rye and sorghum plants. Additionally, we constructed a soil microbial genomic catalog of microorganisms responding to commonly used cover crops, a public resource for agriculturally-relevant microbes. Many of our exudate-stimulated microorganisms are representatives from poorly characterized or novel taxa, highlighting the yet to be discovered metabolic reservoir harbored in agricultural soils. Our findings emphasize the tractability of high-resolution multiomics approaches to investigate processes relevant for agricultural soils, opening the possibility of targeting specific soil biogeochemical outcomes through biological precision agricultural practices that use cover crops and the microbiome as levers for enhanced crop production.

## Background

More than 153,000 U.S. farms currently integrate cover cropping practices on over 15 million acres of land and cover crop adoption has been increasing in recent years [1]. Cover cropping is the incorporation of a secondary, unharvested crop grown typically in rotation with a primary cash crop. Crop diversification through cover cropping influences aboveground plant growth responses and belowground soil characteristics, promoting soil and primary crop health alike [2–1]. For instance, aboveground, cover crops act as a physical barrier against wind and water erosion, protect against weeds, and when utilized to replace periods of fallow, cover crops can contribute to the maintenance of soil functionalities that can bolster primary crop yields [7–10]. Additionally, belowground, cover crops stimulate microbial populations that catalyze beneficial biogeochemical processes [4, 11, 12]. The belowground impact of cover cropping is influenced by both cover crop root exudation during plant growth as well as the cover crop litter as it decomposes, but rarely are these influences detangled [13–15]. Despite the adoption of cover cropping as a regenerative agriculture practice, there is a paucity of data on cover crop root exudate chemical diversity and its impacts on soil biogeochemistry.

Metabolites released by plants as root exudates include diverse sugars, organic and amino acids, enzymes, and secondary plant metabolites. These primarily water-soluble and low-molecular-weight compounds influence the rhizosphere, altering the chemical landscape adjacent to plant roots and shaping the microbial recruitment and associations that are beneficial for plant hosts [16–19]. For example, exuded sugars and amino acids are carbon and nitrogen growth substrates for rhizosphere microorganisms, stimulating microbial activity near roots that provides benefits to the host plant of enhanced nutrient acquisition and pathogen suppression [20–22]. Moreover, secondary metabolites in exudates, like indoles and derivatives, contribute to microbially-mediated plant defense responses, growth, and plant-microbe signaling, playing a crucial role in shaping the overall ecology of the rhizosphere [18, 23, 24]. However, harnessing exudates for targeted biological stimulation of the rhizosphere is complicated by the diversity in both quantity and quality of root exudate compounds resulting from different crop species, and cultivars within species, which is altered in response to growth phase, abiotic and biotic stress, and many edaphic conditions [14, 15, 25–27].

We previously used metabolomics to characterize variability in root exudation across 19 commonly used cover crop species [25]. We assessed primary and secondary root exudates, and importantly, quantified low abundance phytohormones released as exudates. The cover crop species selected here are a subset of this prior study and representative of four functional plant groups (legumes, brassicas, cool-season grasses, warm-season grasses) commonly used in U.S. agriculture [1]. We previously demonstrated these cover crop species had distinct root exudate chemical profiles, a finding we hypothesized could yield distinct soil microbial outcomes.

**Figure 1:**
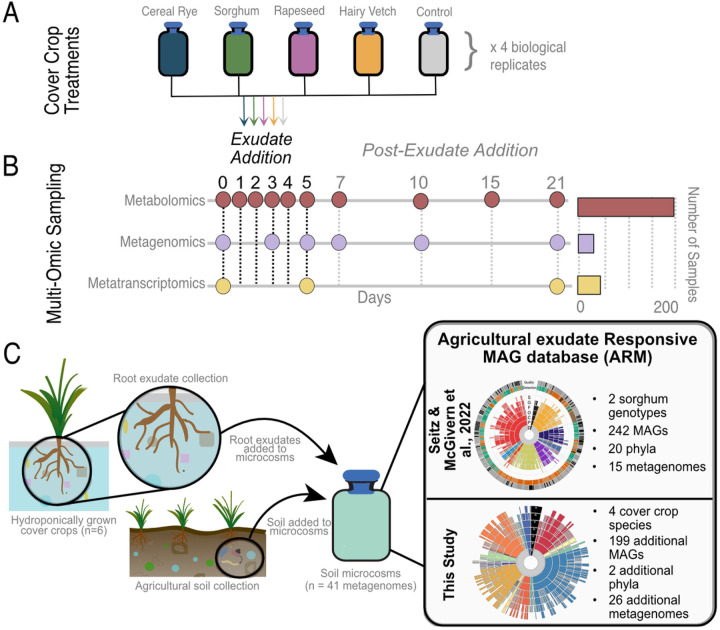
Experimental design. A) Root exudates were collected from 4 hydroponically grown cover crops [25] along with a water control treatment. The soil reactors were amended with soil from the agricultural research station at Colorado State University in biological quadruplets from 5 treatments (i) cereal rye (dark blue), (ii) sorghum (green), (iii) rapeseed (light purple), (iv) hairy vetch (orange) and a water amended soil control (grey). B) In this study, microcosms were amended with root exudates from cover crops as shown in (A) for 6 days (denoted as the exudate addition phase) and responses were surveyed for 21 days (post-exudate addition phase). Metagenomes (n=26), metatranscriptomes (n=40), and metabolomes (n=199) were collected at specific timepoints, indicated by circles, to profile microbial responses. The number of samples collected for each ‘omic measurement is indicated by the bar charts on the right. C) Schematic summary of the data collected for the metagenome assembled genome (MAG) database, which includes content from a prior study [25] and findings reported here. These MAGs were used to construct the Agricultural exudate-Responsive Metagenomic (ARM) database, with contributions from each experiment highlighted by bullet points.

To decipher the microbial responses to cover crop root exudate treatments, we utilized laboratory-scale soil microcosms and stimulated microbial communities with daily amendments of pure root exudates from each over crop species (n = 4) over 6 days, tracking the responses over a 21-day experiment using metabolomics and genome-resolved metatranscriptomics (Figs. 1A–B). We found metabolomics was more sensitive than metagenomics for detecting differences in microbial responses between cover crops treatments and over the timeseries. However, functional gene expression and chemical behavior were still tightly coupled, especially for phytohormone metabolism. We created a microbial genomic resource for exudate-stimulated microorganisms from agricultural soils here referred to as the Agricultural exudate Responsive Metagenomic (ARM) database. This integrated research is a first step towards the development of biological precision agriculture, linking cover cropping microbiome management practices to support valuable ecosystem resources.

## Materials & Methods

### Cover Crop Root Exudate Collections

Mature seeds from the four chosen cover crops *(Sorghum bicolor,* sorghum; *Vicia villosa,* hairy vetch; *Brassica napus,* rapeseed; and *Secale cereal,* cereal rye) were first surface sterilized in a sterile tissue culture hood by placing seeds in 15 mL conical tubes with (A) 2 mL of Clorox bleach solution (3% sodium hypochlorite. 1:1 bleach to water) +1 drop of Tween-20 to reduce surface tension and improve sterilization or (B) 2 mL of Clorox bleach solution (3% sodium hypochlorite. 1:1 bleach to water) (see **Additional File 1** for sterilization optimization). Sterilization method A or B was chosen based on optimization of germination rate for each cover crop species. Seeds suspended in sterilization solution were shaken for 1 min. In a sterile tissue culture hood, bleach was removed using sterile techniques and the seeds were rinsed five times with sterile deionized (DI) water. Seeds were either (A) plated to agar plates with MS basal salt mixture (2.16 mg Murashige and Skoog (MS) media in 500 mL sterile DI water; MP Biomedicals, Santa Ana, CA) germinated in the dark for 3 days, and then transferred to sterile tubes with 3 mL liquid MS media after 3 days; or (B) placed directly in sterile growth vessel with 3 mL MS media. Growth vessels were either glass tubes which were used for monocotyledon species (cereal rye and sorghum) or Magenta boxes which were used for dicotyledon species (rapeseed and hairy vetch) based on preliminary experiments to optimize growth of each seedling. All seeds were incubated in a growth chamber with photoperiod 16 h light/8 h night at 25°C for 14 days **(Additional File 1).** The 14-day-old seedlings utilized did not introduce any microbes to the system as no bacterial growth was observed on the MS agar plates or growth containers. Cover crop seedlings were grown for 14 days before root exudate collections. Root exudates were collected by first rinsing the roots in sterile DI water 3 times and transferring to a new, sterile vessel filled with 5 mL sterile DI water for a 24 hr root exudate collection period. The suspensions containing the root exudates were then filtered through a 0.2 μm filter membrane to remove root detritus. Enough root exudates were collected and pooled together to achieve a treatment addition of 1.5 mg of pure exudates per day (36 mg total needed for experiment). 1.5 mg of exudates was chosen based on the average amount of exudate mass exuded per day across crop species [25]. Root exudates suspensions were frozen at −20°C in 200 mL sterile glass jars. Frozen root exudate samples were lyophilized completely (~ 72 h), then weighed before resuspension in sterile HPLC-grade water (Thermo Fisher Scientific, Waltham, MA). Resuspended exudates were vortexed thoroughly to remove all residue from the bottle (1min of vortexing along all edges of the bottle). A volume equivalent to 1.5 mg was aliquoted into new sterile glass jars for daily addition during the experiment and frozen at −20°C until use.

### Cover Crop Root Exudate Microcosm Experimental Setup

Microcosms were established and sampled as previously described [28, 29] and metadata collection of all samples follows the recommended standards for agricultural microbiome research [30]. The soil (microbial inoculum) was collected from an agricultural field at the Colorado State University Agricultural Research and Education Center near Fort Collins, CO on June 21, 2022 (GPS coordinates: 40.65493226483286, −104.99860614985835). The climate at the site is semi-arid, with 408 mm mean annual precipitation and a mean annual temperature of 10.2°C (1981–2010 average, https://usclimatedata.com/). The soil is classified as an Aridic Haplustalf. Three 2-cm diameter soil core samples at approximately 12 cm depth were collected from 5 random locations within the plot. The soil was stored on ice during transportation and at 4 °C in the laboratory until microcosm construction the following day. 20 g of soil from each location within the plot was pooled, homogenized, and sieved (2 mm) to create a representative bulk soil repository used in the microcosms experiment. 4 g of homogenized soil and 45 mL of sterilized water were added to quadruplet sterile 100-mL glass bottles to construct each microcosm (n = 20). Microcosm slurries were vortexed and allowed to settle for 5 min, with day 0 samples then taken by removing 3 mL of soil slurry for parallel microbiome analysis. The microcosm slurry samples were centrifuged at 10,000 rcf for 10 min, with the supernatant used as metabolome sample and the pellet used in the RNA/DNA extraction. After baseline sampling (day 0), 4 mL of exudate treatment (1.5 mg pure, dried, root exudates in 4 mL sterilized LC-MS-grade water) was added to each microcosm replicate and briefly vortexed. At this point, for each microcosm the bottle caps were removed and replaced with a sterilized foam stopper (model number: Outus-17731) for the rest of the experiment to maintain oxic conditions and prevent colonization by contaminating microbes. Microcosms were incubated in an orbital shaker set at 200 rpm at 24°C for 21 days. Each exudate treatment (1.5 mg exudate/4 mL water) (Fig. 1A) was added to microcosms on days 0, 1, 2, 3, 4, and 5 at approximately the same time every day. After day 5, no additional exudate treatments were applied, but microcosms were maintained until day 21, which afforded additional samples taken at days 7, 10, 15, and 21 (Fig. 1B). Samples were collected (3 mL of soil slurry removed and centrifuged to obtain pellet for DNA/RNA and supernatant for metabolome) at roughly the same time each day and collected with aseptic techniques to ensure no additional microbial influence was introduced. Samples for metabolomics and DNA/RNA were immediately frozen at - 80°C until processing.

### Metabolomics: Sample Preparation

Metabolomic analyses (targeted UPLC-MS/MS and nontargeted LC-MS/MS) was performed on all microcosm treatments (n = 5) across each of the ten timepoints (days 0, 1, 2, 3, 4, 5, 7, 10, 15, 21, n = 10) in quadruplicate (total n = 200 samples per analysis type). Once ready for analysis, metabolomic samples were lyophilized and weighed to record the sample mass (tubes were pre-weighed prior to the experiment in order to calculate mass). We then added 1 mL sterile HPLC-grade water to each sample, and a volume equivalent to 0.25 mg of sample was transferred to a new, 2 mL glass autosampler vial and re-lyophilized to create two 0.25 mg subsamples for targeted and non-targeted metabolomics.

### Metabolomics: Targeted UHPLC-MS/MS for Phytohormone Analysis

To measure low abundance phytohormones, the first 0.25 mg subsample was extracted in 75 μL of a spiked methanol solution containing 100% methanol with 65.2 ng/mL abscisic acid-d6, 62.5 ng/mL salicylic acid-d6, and 90.0 ng/mL jasmonic acid-d5 (Sigma). After solvent addition, samples were placed on a shaker plate for 1 hr at the highest speed setting, centrifuged at 3500 × g at 4°C for 5 min, and transferred to glass inserts. A final centrifuge step at 3500 × g for 15 min at 4°C was completed to ensure any precipitate was in the bulb of the vial insert. To measure instrument function a pooled QC was creating by combining 5uL of each sample to a separate vial. This pooled QC was run after every 6 injections. Five microliters of extract was injected onto an LX50 UHPLC System, equipped with an LX50 Precision Sampling Module (20-μL sample loop, partial loop injection mode) (PerkinElmer, Waltham, MA, USA). An ACQUITY UPLC T3 column (1 × 100 mm, 1.8 μM; Waters Corporation) was used for chromatographic separation. Mobile phase A consisted of LC-MS grade water with 0.1% formic acid and mobile phase B was 100% acetonitrile. Elution gradient was initially 0.1% B for 1 min, which was increased to 55.0% B at 12 min and further increased to 97.0% B at 15 min, then decreased to 0.1% B at 15.5 min. The column was reequilibrated for 4.5 min for a total run time of 20 min. The flow rate was set to 200 μL/min and the column temperature was maintained at 45°C. Samples were held at 4°C in the autosampler. Detection was performed on a QSight^™^ 420 triple quadrupole mass spectrometer (MS) operated in selected reaction monitoring (SRM) mode. SRM transitions for each compound were optimized through analysis of authentic standards **(Additional File 2).** The MS was operated with the ESI voltage 5000 V in positive mode and - 5000 V in negative mode. Nebulizer gas flow was set at 350 arbitrary units and drying gas was set to 120 arbitrary units. The source temperature was 315°C and hot-surface induced desolvation temperature was 200°C.

### Metabolomics: Nontargeted LC-MS and fastDDA Analysis

To broadly measure primary and secondary metabolites, the second 0.25 mg subsamples were extracted in 80 uL of 20% MeOH, sonicated for 1h at 20°C, centrifuged at max speed for 15 min, and transferred to glass inserts for analysis. From each sample, 5 uL was aliquoted into a separate vial to be used as a pooled QC to monitor proper instrument function and to detect any analytical variation. From each sample within a treatment, 5 uL was aliquoted into a 2 mL vial to be used as a treatment pool for fastDDA.

One microliter of each sample was injected onto a Waters Acquity UPLC system. Separation was achieved using a Waters ACQUITY UPLC Premier BEH Amide 1.7 μm Column (2.1 × 100 mm), using a gradient from solvent A (10 mM ammonium formate in water with 0.125% formic acid) to solvent B (95:5 acetonitrile:water, 10 mM ammonium formate in water with 0.125% formic acid) and a flow rate of 0.5 mL/min. Column eluent was infused into a Waters Xevo G2-XS Q-TOF-MS with an electrospray source in positive ion, sensitivity mode, with data dependent acquisition (DDA). For individual samples, the following parameters were used for MS1 survey scan: 50–1200 m/z at 0.2 sec per scan, switching to MS/MS after individual ion intensity rises above 10000. MS/MS acquisition occurred at a scan rate of 0.2 sec, or at accumulated TIC threshold of 100000, with 1 MS/MS event per MS1 scan. No inclusion list was used, but dynamic peak exclusion was used with a 30 second and 100 ppm mass difference exclusion window. A pooled QC standard was run after every 7 normal sample injections.

In addition, pooled cereal rye, rapeseed, sorghum, control soil, control water, and hairy vetch samples were run separately with 10 replicate injections per pool in iterative exclusion mode using AutoCat_V1 processing. In this case the following parameters were used for MS1 survey scan: 50–1200 m/z mass range at 0.1 sec per scan, switching to MS/MS after individual ion intensity rises above 5000. MS/MS acquisition occurred at a scan rate of 0.5 sec, or after accumulated TIC threshold of 100000, with 5 MS/MS event per MS1 scan. For all experiments, collision energy for MS/MS was ramped from 15 to 30 V. Calibration was performed using sodium formate with 1 ppm mass accuracy. The capillary voltage was held at 700 V in positive mode. The source temperature was held at 150°C and the nitrogen desolvation temperature at 450°C with a desolvation flow rate of 1000 L/hr. Lockspray reference mass was used to correct for drift, with 40 sec interval between scans, 0.1 sec/scan and signal averaged over 3 scans. LeuEnk was used for mass correction, with reference mass of 556.2771 m/z. For pooled iterative exclusion samples, lockspray signal was collected but correction was not applied until post-processing. These latter data were processed using Waters Tynebridge to produce mass calibrated mzML files compatible with GNPS server. The column and samples were held at 30°C and 6°C, respectively.

### Metabolomics: Data Analysis

For the analysis of low abundance phytohormones, Simplicity 3Q software (Version 3.0.2, PerkinElmer, Waltham, MA) was used to detect and integrate peak areas and to a calculate linear regression of analytical standards used for quantification. Each peak was normalized to an appropriate internal standard. The corresponding linear regression equation was used for quantification (ng/mL) for each analyte, which was then adjusted for precise volume of sample (ng/mL). The limit of detection was calculated as 3 times the standard deviation of the blank divided by the slope of the calibration curve. Likewise, the limit of quantitation was calculated as 10 times the standard deviation of the blank divided by the slope of the calibration curve.

For non-targeted LC-MS data, mzML files were processed through the following workflow: 1) XCMS software was used for preprocessing to identify molecular features [31]; 2) features were further normalized to total ion current; 3) the package RAMClustR [32] was used for clustering features into spectra. MSFinder [33] was used for spectral matching, formula inference, and tentative structure assignment.

For LC-MS/MS fastDDA data, mzML files for each iterative exclusion across all treatments (n = 50) were uploaded to GNPS for molecular networking and annotation. A molecular network was created using the online workflow (https://ccms-ucsd.github.io/GNPSDocumentation/) on the GNPS website (http://gnps.ucsd.edu). The data was filtered by removing all MS/MS fragment ions within +/−17 Da of the precursor m/z. The precursor ion mass tolerance was set to 0.5 Da and a MS/MS fragment ion tolerance of 0.02 Da. A network was then created where edges were filtered to have a cosine score above 0.5 and more than 5 matched peaks. Further, edges between two nodes were kept in the network if and only if each of the nodes appeared in each other’s respective top 20 most similar nodes. Finally, the maximum size of a molecular family was set to 100, and the lowest scoring edges were removed from molecular families until the molecular family size was below this threshold. The spectra in the network were then searched against GNPS^’^ spectral libraries. The library spectra were filtered in the same manner as the input data. All matches kept between network spectra and library spectra were required to have a score above 0.5 and at least 4 matched peaks.

Annotation of compounds to the level 2 classification was completed through manual matching between the MS1 data from all samples and the MS2 data collected from the pooled QCs. Specifically, annotation results from GNPS molecular networking were exported and precursor masses were matched to MS1 spectra within an 11s retention time window (to account for analytical drift). Quantitative data (untargeted LC-MS and targeted UHPLC-MS/MS for phytohormone analysis) were z-scored and combined for the final quantitative dataset **(Additional File 2).**

### Metabolomics: Chemical Taxonomic Assignments

For nontargeted LC-MS/MS molecular features, MSFinder InChiKey results were exported. For targeted LC-MS/MS, InChiKeys were gathered from PubChem. All InChiKeys were uploaded to ClassyFire for batch annotation of chemical taxonomy [34]. Metabolites quantified via targeted UHPLC-MS/MS were assigned a level 1 annotation. A level 2 annotation was assigned to molecular features in which a feature in MS1 was also present in MS2. A level 3 annotation was assigned to molecular features that did not have corresponding MS1 and MS2 data, but could be assigned chemical taxonomy according ClassyFire.

### Metagenomics: Sample Preparation

For days 3, 5, 7, 10, and 21, we obtained a metagenome for each treatment from a single sample (n = 25 metagenomes; Fig. 1B). For day 0, the control microcosm was used for a total of 26 metagenomes sequenced. For these samples, genomic DNA was prepared for metagenomic sequencing using the ZymoBiomics DNA/RNA Miniprep kit and the Tecan Ovation Ultralow System V2 library prep kit and was sequenced at the University of Colorado Anschutz Sequencing Core on the Illumina NovaSeq 6000 with 2×150 bp chemistry at 20 million read pairs per sample. This resulted in an average of 9.12 Gbp per sample, for a total of 237.15 Gbp of metagenomic sequencing.

### Metagenomics: Assembly, Binning, and Annotation

Fastq files were trimmed using Sickle (v1.33) [35], and trimmed reads were assembled using MEGAHIT (v1.2.9) [36]. To maximize genome recovery, we performed 3 assemblies: (1) individual assembly on each fastq (n = 26), (2) co-assembly by combining reads from each treatment fastq metagenomes to increase assembly coverage (n = 5) and (3) a single iterative assembly was performed on reads that did not map to binned assembled scaffolds ≥ 2.5 kb at 97% identity on all metagenomes. Information for metagenome statistics, including assembly information, are found in **Additional File 3.** For each assembly, scaffolds ≥ 2.5 kb were binned using MetaBAT2 (v2.12.1) [37], and MAG quality was assessed using checkM (v1.1.2) [38]. MAGs were kept in the database if they were > 50% complete and < 10% contaminated. The resulting 441 MAGs were dereplicated at 99% identity using dRep in 326 MAGs (v2.6.2) [39]. MAG taxonomy was assigned using GTDB (v2.3.0 Release 08-RS214) [40]. MAGs and assemblies were annotated using DRAM (v1.4.4) [41]. CAZymes were inferred from the DRAM hits. See **Additional File 4** for DRAM distillate and **Additional File 5** for raw annotations. See **Additional File 6** for ARM database fasta.

To quantify MAG relative abundance in each metagenome, trimmed metagenomic reads were mapped to the dereplicated MAG database using Bowtie2 (2.4.5), output sam files were converted to sorted bam files using samtools (v1.9) [42] and entries in the bam file were filtered to 95% identity using reformat.sh from BBMap at minidfilter = 95 [43]. We had two requirements for a MAG to be present in a sample: first we required reads to map to at least 75% of a MAG in a given sample, and second the MAG had to have at least 3X coverage in that sample. To determine MAGs that had reads mapped to at least 75% of the MAG, we used coverM (v0.6.0) [44] in genome mode to output MAGs that passed this threshold (-min-covered-fraction 75). To obtain MAG coverage, we used coverM (v0.3.2) in genome mode to output reads_per_base (reads mapped/genome length), and from this calculated MAG coverage as reads_per_base × 151 bp. A bin was present in a treatment or in control if it was found in any of the timepoints.

### Metagenomics: Gene Annotation Curation

Genes for cysteine desulfhydrase are known to misannotate as 1-aminocyclopropane carboxylic acid (ACC) deaminase (*acdS)* [45–47]. To curate these, we took genes that were annotated as “K01505” from DRAM and aligned them against a known and validated *acdS* (UniProt: Q5PWZ8) using MUSCLE (default parameters) [48]. We then looked for the key *acdS* active site residues E295 and L322, and confirmed the presence of K51, Y268, and Y294 **(Additional File 3).** Genes for gibberellic acid (GA) biosynthesis in KEGG are annotated for fungi and plants and do not annotate model microorganisms that have GA biosynthetic capacity. Instead, we used a BLAST analysis with individual protein sequences of each gene as the query to survey MAGs containing genes located in the GA operon [49]. MAGs that contained sequence matches were considered if the BLAST E value was 10^−10^ or less and a bitscore >150. Because *CYP115* is the key enzyme converting GA_9_ to GA_4_ this was used as the determinate for a MAG that produces bioactive GA_4_. Preliminary nitrogen oxidation and reduction DRAM annotations were confirmed using a combination of phylogeny and active site analysis where appropriate.

### Metatranscriptomics: Sample Preparation

For days 0, 5, and 21, we obtained triplicate metatranscriptomes for each treatment/timepoint. However, library preparation failed for replicate 2 of each treatment at day 5 leading to n = 40 metatranscriptomes (Fig. 1B). RNA was prepared for sequencing using the ZymoBiomics DNA/RNA Miniprep kit and cleaned using ZymoBiomics RNA Clean & Concentrate Kit and sent to the Department of Energy’s Joint Genome Institute for sequencing on the Illumina NovaSeq 6000 with 2×151 bp chemistry at a target depth of 100M reads per sample. rRNA was depleted from an input of 10 ng of total RNA using QIAseq FastSelect^™^ - 5S/16S/23S, rRNA Plant and rRNA Yeast Kits (Qiagen). Using TruSeq stranded mRNA kit (Illumina), the 300 bp - 400 bp heat fragmented RNA was reverse transcribed to create the first strand of cDNA with random hexamers and superscript^™^ II Reverse Transcriptase (Thermo Fisher Scientific) followed by second strand synthesis. The double stranded cDNA fragments were treated with A-tailing, ligation with JGI’s unique dual indexed adapters and enriched using 13 cycles of PCR.

### Metatranscriptomics: Mapping and Analysis

Fastq files were trimmed, and adapters were removed using bbduk (v38.90) with the parameters ktrim = r, k = 23, mink = 11, hdist = 1, and filtered using rqcfilter2 (v38.90). Trimmed, filtered reads were then mapped via Bowtie2 [50] to the MAG database (dereplicated to 99% ID). Sam files were transformed to bam files using samtools, filtered to 95% id using reformat.sh and name sorted using samtools. Transcripts were counted for each gene with feature-counts [51]. Genes were considered if they had counts greater than or equal to 3 in at least 3 samples. The counts were then transformed to geTMM (gene length corrected trimmed mean of M-values) in R using edgeR package [52]. MAGs were considered present in a sample if they had greater than or equal to 10 genes in a sample **(Additional File 7).** We also analyzed the metatranscriptome at the function level, where KEGG, MEROPS, and dbCAN2 assigned annotations were used to infer gene function. Gene geTMMs within a function were summed to obtain the overall functional activity of the microbial community within a metatranscriptome **(Additional File 7).**

### Statistics

Peak area files for each metabolomic analysis were combined into one file **(Additional File 2)** and normalized by z-scoring. Univariate statistics were conducted in GraphPad Prism (v9.4.1). Line graphs of targeted and nontargeted data were visualized in Prism using absolute concentrations and TIC normalized values, respectively. Two-way ANOVAs (multiple comparisons were corrected using the recommended multiple comparisons test) were calculated using log_10_ transformed data to satisfy the assumption of data normality in order to determine if a compound was significantly different between a treatment and the control at each timepoint. Multivariate metabolomic statistics were conducted in SIMCA (v17.0.1) to generate partial least square discriminate analysis (PLS-DA) models and gather variable importance in projection (VIP) scores (here, we used a cutoff of VIP > 1.8). PLS-DA was performed using annotated and unannotated metabolites using z-scored and UV-scaled data in SIMCA. The list of scores and loadings coordinates were then plotted in Prism. Significance between metabolic changes across time and treatment was quantified using the adonis2 commands from the vegan package in R (v4.3.1) with a PERMANOVA model [53]. Pairwise comparisons were completed using the pairwise_permanova commands.

Metatranscriptomic non-metric multidimensional scaling (NMDS) plots were generated in R (v4.3.1) to estimate beta diversity across treatments. We utilized Bray-Curtis dissimilarity matrix visualized by NMDS in R with the ggplot2 package with stress of the non-parametric fit for the goodness of fit for the model [54]. NMDS scores were exported from R and imported for visualization into Prism. Significance of compositional differences across treatments and the interaction of treatment and time, was quantified using a multi-response permutation procedure, and the betadisper commands from the vegan package (v2.6–4) with an ANOVA model in R [53]. Metatranscriptomic hierarchical clustering was completed in R using the hclust function from the stats package (v4.3.1). Visualization of the clustering used the as.dendrogram function in the stats package.

Discriminant metatranscriptome MAGs and functions were identified using MaAsLin2 [55] using default parameters with various tests considered: 1) day 5, MAG-level expression, control versus each individual treatment, 2) day 5, MAG-level expression, control versus all exudate treatments, 3) day 5, function-level expression, control versus all exudate treatments. All MaAsLin2 analyses were ran in R using the treatment (control or exudate) as a fixed effect. See **Additional File 8.**

**Figure 2:**
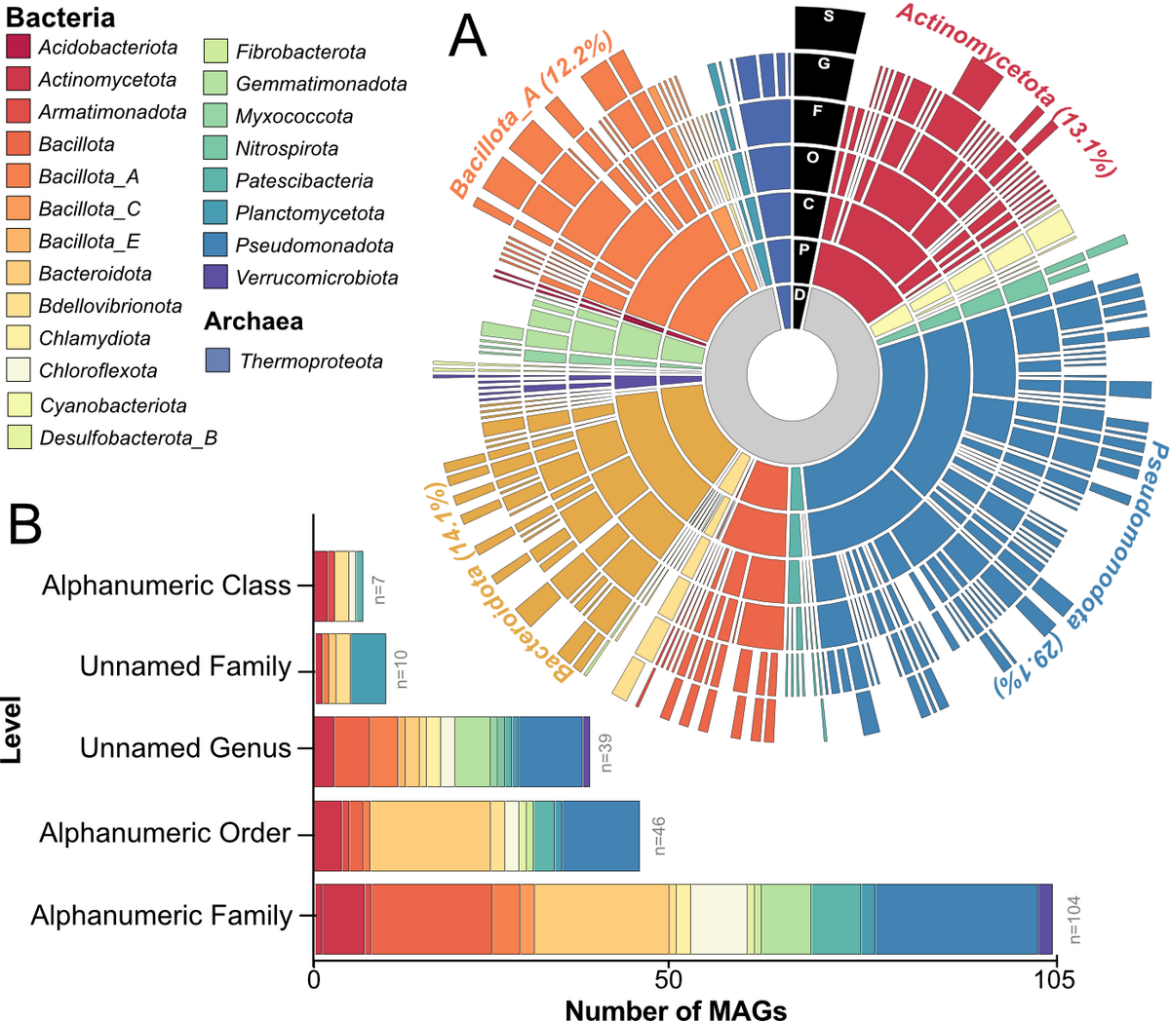
Curation of an exudate-responsive metagenome assembled genome (MAG) database. A) The taxonomy of the dereplicated MAGs are shown by sequentially colored rings ordered from domain (D, inner ring; grey=bacteria, purple=archaea), phylum (P), class (C), order (O), family (F), genus (G), to species (S, outer ring) assignment. Ring color corresponds to phylum, with the taxonomic assignment denoted in the legend to the left. Gaps at each level represent MAGs that were unclassified at that level of taxonomy (according to GTDB v2.3.0 08-RS214). B) Stacked bar chart shows novelty of ARM MAGs when compared to GTDB. Bars indicates the number of dereplicated MAGs recovered that represent unassigned families and genera as well as MAGs assigned only an alphanumeric identifier. Here, novelty is defined as the first unnamed level (i.e., unnamed family or genus) and the level where an alphanumeric identifier is used (i.e., alphanumeric family). Coloring corresponds to MAG phylum.

**Figure 3:**
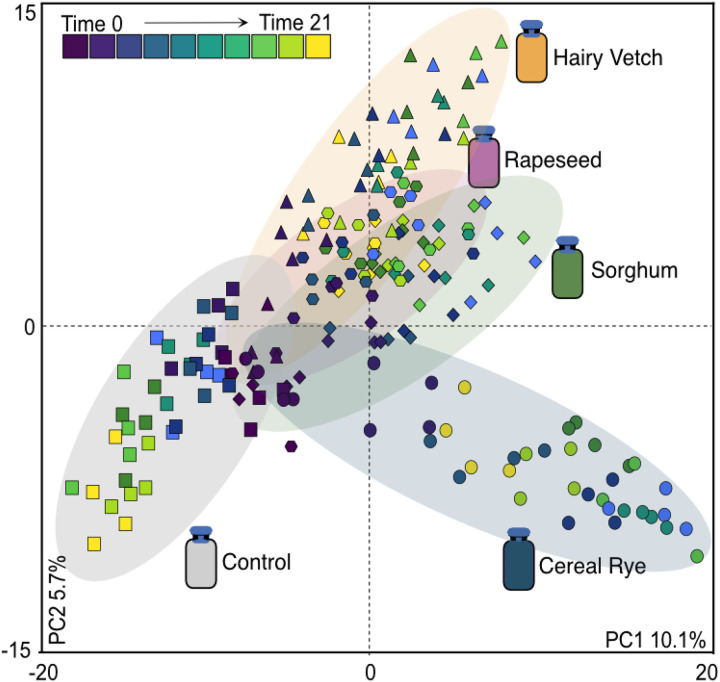
Cover crop exudate treatments influence soil microbial metabolomes. Two-dimensional scores plot of partial least squares discriminate analysis (PLS-DA) (R_2_X = 0.101, R_2_Y = 0.0597, Q_2_ = 0.784, PERMANOVA, p<0.05) between all treatment metabolomes. Each point represents a metabolome, with colors representing time. Shapes denote treatment: cereal rye (circles), hairy vetch (triangles), sorghum (diamonds), octagons (rapeseed) and control (square) metabolomes. Ellipses represent 95% confidence intervals and are colored by treatment. Corresponding loadings biplot can be found in **Additional File 9**, Figure 2.

**Figure 4:**
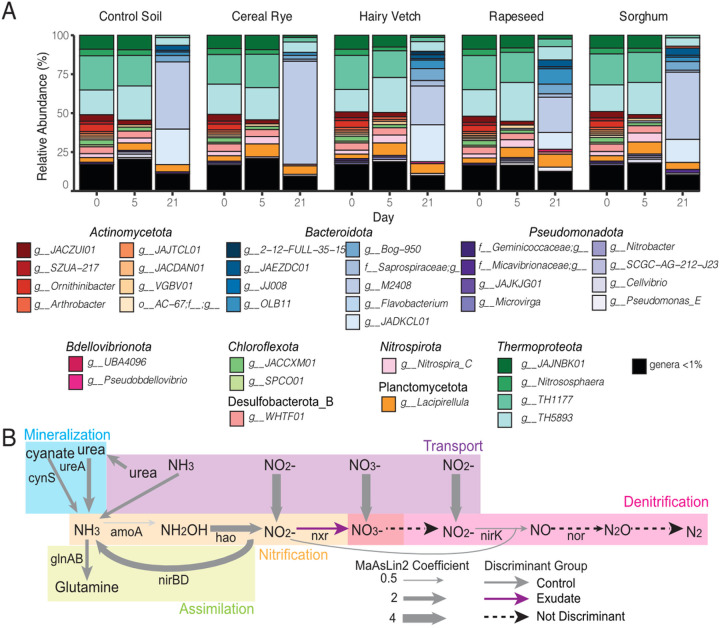
Overall expressed gene content but not transcriptionally active genera, are altered by cover crop exudates. (A) Average metatranscriptome profiles of the MAGs from control and exudate microcosms. MAG metatranscript abundance was summed at the genus level and then averaged across replicates. Colors correspond to MAG genus and MAGs with an abundance less than 1% are shown in black. (B) Nitrogen cycling functions differed between control and all cover crop exudate metatranscriptomes at day 5. Arrow thickness corresponds to significant MaAsLin2 coefficients (roughly effect size), and arrow color indicates the treatment with which the feature associated (grey for the control, purple for the exudates). Dashed black lines correspond to functions that were detected in the metatranscriptomes but were not discriminant. For plots of nitrogen function expression, see **Additional File 9, Figure 9.** To see all discriminant functions and their statistics, see **Additional File 8.**

**Figure 5:**
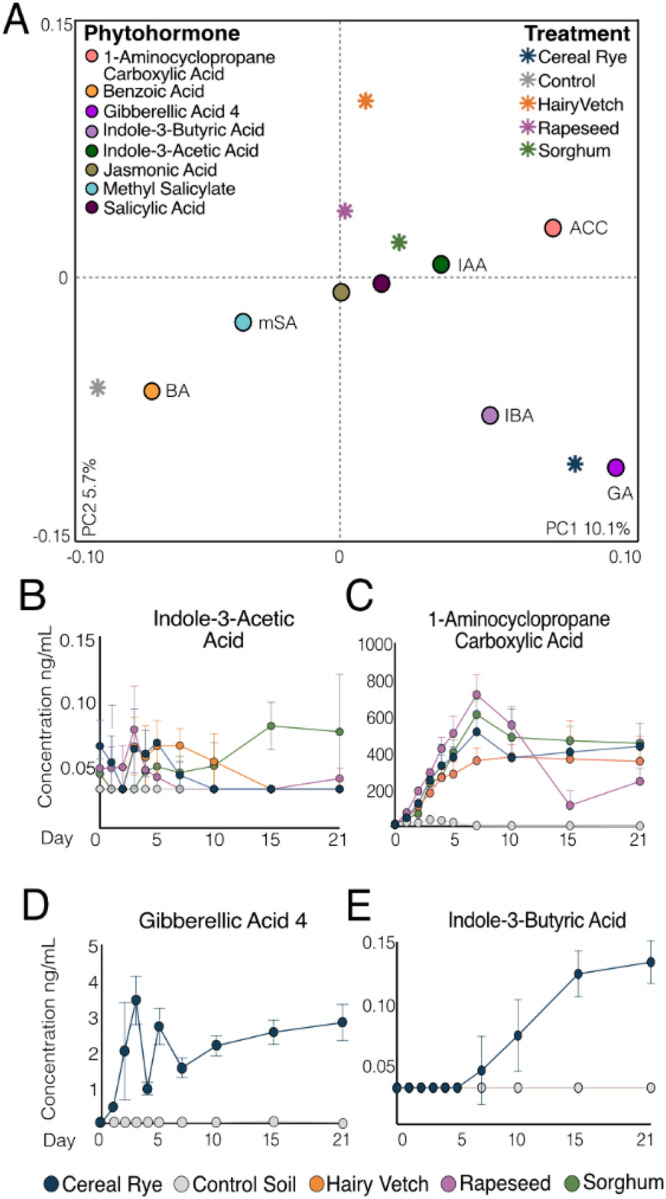
Cover crop treatment influences microbial phytohormone biosynthesis across time. A) Partial least squares discriminate analysis (PLS-DA) (R_2_X = 0.101, R_2_Y = 0.0597) biplot shows the relative contribution of each phytohormone abundance found within a treatment metabolome. Colored stars indicate a treatment and colored circles indicate a phytohormone. Colored circles with text labels indicate phytohormones discussed in the text. B-D) Line graphs show temporal dynamics of 4 phytohormones colored by treatment. Circles represent the average concentration at that timepoint, and error bars represent one standard deviation. (B) Indole-3-acetic acid (IAA) (C) 1-aminocyopropane carboxylic acid (ACC) (D) Gibberellic acid 4 (GA_4_) (E) Indole-3-butyric acid (IBA).

**Figure 6:**
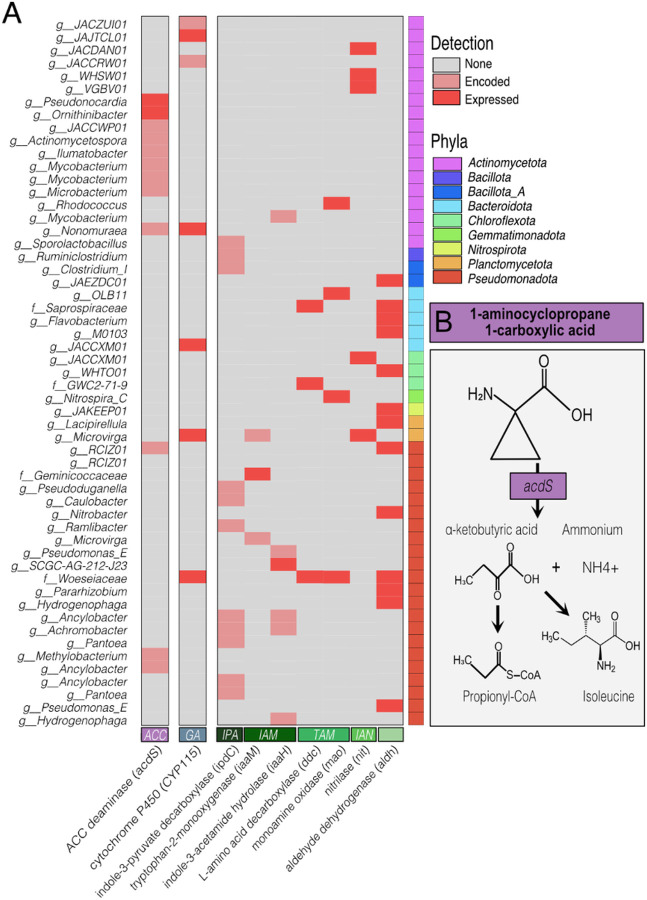
Gene potential and expression across phytohormone metabolic routes. A) heatmap shows the expression (in red) or the gene potential (in pink) for each gene in (i) ACC degradation (*acdS*; purple), (ii) GA_4_ biosynthesis (*CYP115;* blue), or (iii) IAA biosynthesis (shades of green correspond to different IAA pathways; darkest green for the indole-3-pyruvate (IPA) pathway, dark green for the indole-3-acetamide (IAM) pathway, green for the tryptamine (TAM) pathway, and light green for the indole-3-acetonitrile (IAN) pathway, the final lightest shade of green represent the terminal oxidation of indole acetaldehyde (IAAId) to IAA)). MAGs listed are those only detected in exudate amended microcosms and not controls as indicated in the main text. When genus name was undescribed, family name was used. Colored boxes to the right indicate MAG phylum with taxonomy in the legend to the right. Colored boxes at the bottom indicate the pathway. B) ACC deamination *(acdS)* yields ammonium and α-ketobutyric acid, a compound which can be further transformed to propionyl-CoA or isoleucine **(Additional File 3).**

**Figure 7:**
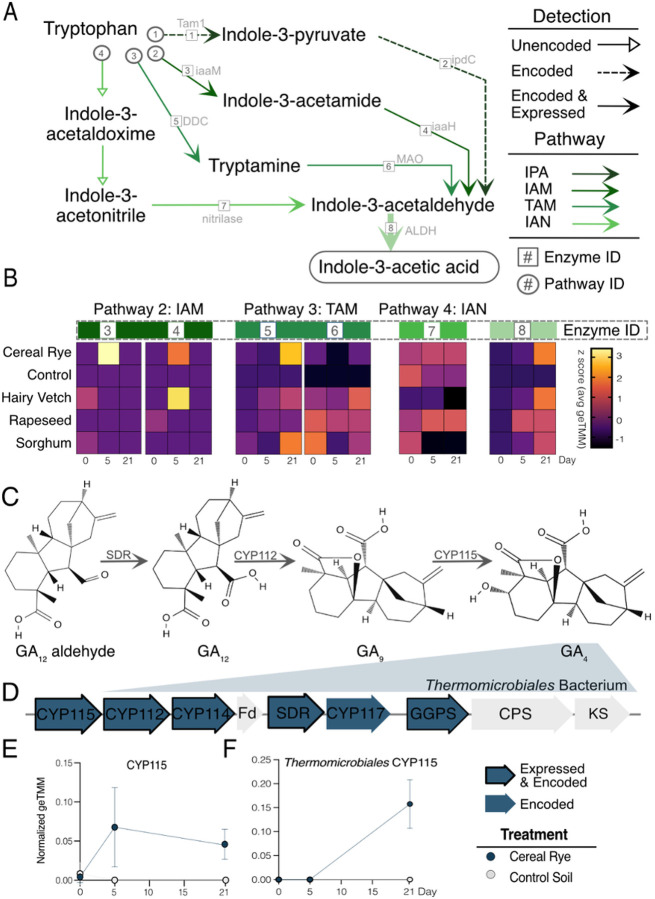
Indole-3-acetic acid and gibberellic acid 4 biosynthesis routes and associated MAGs. A) IAA biosynthetic pathways. Four routes for IAA biosynthesis were detected by metagenomics and/or metatranscriptomics where detection is indicated by arrow type: hollow arrows indicate the gene was not detected, dotted arrows indicate the gene was only encoded (metaG only), solid arrows indicate the gene was encoded and expressed. Enzymes names are in grey next to the corresponding reaction arrow and given an enzyme number (“enzyme #”). Pathways are given a corresponding ID # in circles. Colors correspond to IAA pathway. B) Heatmaps show the z-scored geTMM value for each summed gene abundance across all MAGs expressing the gene (corresponding to the enzyme number in (A)) within a treatment and timepoint. C) Downstream GA_4_ biosynthetic route for production of bioactive GA_4_. D) The GA operon highlighting a potentially-new bacterium of the *Thermomicrobiales,* which expressed 5 of 9 GA biosynthetic proteins required to produce GA_4_, including the final enzyme converting GA_9_ to GA_4_. Grey arrows indicate the gene was neither expressed or encoded in the *Thermomicrobiales* MAG, blue arrows show an encoded gene, and a blue arrow with a black border show a gene was encoded and expressed in the *Thermomicrobiales* MAG. E) Averaged geTMM abundance of MAGs producing *CYP115* protein in cereal rye and control microcosms. F) Expression of *CYP115* from a novel *Thermomicrobiales* bacterium protein in cereal rye and control microcosms

Figure 1 was created from scratch in Affinity Designer (v2.3.0). Figures 2–7 were created using a combination of GraphPad Prism (see above methods) and RStudio generated figures (see GitHub link, available in the data availability section, for R code). All final figures were curated in Affinity Designer.

## Results

### Agricultural Exudate- Responsive Metagenomic (ARM) Database Expands Microbiome Knowledge in Colorado Soils

To understand how microbial genomic content influences soil health and the promotion of plant growth in response to exudate stimulation, we established the Agricultural exudate-Responsive Metagenomic (ARM) microbial genomic catalog. In total, we collected 41 metagenomes from two exudate stimulation soil microcosm experiments (Fig. 1C), resulting in 621 Gbp of total metagenomic sequencing. The prior proof of concept study evaluated sorghum genotype exudate profiles using microcosms operated similarly except exudate profiles guided the formulation of synthetic root exudates [28], while here we directly added the cover crop produced exudates to the soil microcosms. From both experiments we reconstructed 441 medium- and high-quality metagenome-assembled genomes (MAGs) that were dereplicated at 99% identity into 326 MAG clusters, representative of distinct microorganisms (Fig. 2A). ARM contains MAGs assigned to 21 bacterial phyla and 1 archaeal phylum, with the database composed mainly of members of *Pseudomonadota* (29.1%), *Bacteroidota* (14.1%), *Actinomycetota* (13.1%), and *Bacillota_A* (12.2%). The MAGs of ARM span 43 different genera **(Additional File 9,** Fig. 1).

Highlighting the genomic novelty of these soils, the ARM database contains MAGs that represent previously unidentified (i.e., lacking a taxonomic assignment) family (n = 10 MAGs, 3%) and genera (n = 39 MAGs, 11.9%) in the Genome Taxonomy Database (GTDB) (Fig. 2B, **Additional File 3).** Additionally, a large proportion of our MAG database belonged to lineages only recognized by alphanumeric identifiers (e.g., Draft Genome Sequence) in GTDB at the class (n = 7), order (n = 46), and family (n = 104) levels (Fig. 2B). Together these findings emphasize the phylogenetic novelty yet to be genomically captured from agricultural soils. Ultimately, accessible ARM resources provide new genomic information for ecologically relevant taxa, with the goal towards enabling taxonomic analyses and metabolic reconstruction of microorganisms from agricultural soils.

### Cover crop exudates results in distinct microbial chemical landscapes

We first examined how soil microbial metabolite pools changed in response to the addition of cover crop exudates over time. Combining non-targeted and targeted LC-MS/MS data resulted in the detection of 641 molecular features to resolve community metabolic changes across the enrichment timeseries in each treatment **(Additional File 2; Additional File 9,** Fig. 2). The metabolite data showed restructuring by cover crop exudate treatment and time (Fig. 3 & **Additional File 9,** Fig. 3 & **Table 1).** Additionally, the cereal rye and hairy vetch treatments exhibited significantly different metabolomes from the control at each matched timepoint following the first day of exudate addition, indicating adding exudates not only rapidly influenced microbial outputs during the time they were added, but had an impact on restructuring of metabolic outputs after the amendment period. In the sorghum and rapeseed exudate treatment, the exudate amendments only differed from control on days 3 and 5, respectively, of the amendment period, yet despite these slight chemical changes early on, they did result in a temporally significant trajectory different from the control later in the timeseries **(Additional File 2).** These results suggest that that cereal rye and hairy vetch root exudates may have more distinct chemical influence over the soil microbiome than sorghum and rapeseed treatments.

The 641 detected metabolites were then classified into 14 superclasses across all samples: lipids and lipid-like molecules (n = 179), organoheterocyclic compounds (n = 106), organic acids and derivatives (n = 97), phenylpropanoids and polyketides (n = 64), organic oxygen compounds (n = 52), benzenoids (n = 38), nucleosides, nucleotides, and analogues (n = 20), alkaloids and derivatives (n = 18), organic nitrogen compounds (n = 15), lignans, neolignans and related compounds (n = 7), organosulfur compounds (n = 3), organic 1,3-dipolar compounds (n = 1), organic polymers (n = 1) **(Additional File 9,** Fig. 4; **Additional File 2).** We found a significant difference between treatment and control metabolomes at this metabolite classification level **(Additional File 9,** Fig. 5 & **Table 2).** Specifically, all exudate-treated microcosms were significantly enriched for lipids, benzenoids, alkaloids and derivatives, and organoheterocyclic compounds. Water amended soil controls were enriched for organic acids and derivatives, nucleosides, nucleotides, and analogues, and organic polymers **(Additional File 9, Table 3).** Interestingly, cereal rye was significantly different from the control and other exudate treatments being enriched for metabolites from the phenylpropanoids and polyketides superclass **(Additional File 9, Table 3).** Taken together our broad metabolite profiling indicates that cover crop exudates can result in distinct chemical trajectories, possibly through altered microbial metabolism.

### Microbial Community Function is Altered by Exudate Addition

Next, we wanted to examine the microbial community response to exudates at the genome membership and gene expression levels. We used the ARM database to resolve metatranscriptome expression profiles across the enrichment timeseries **(Additional File 7).** Unlike the metabolomes that had a strong individual cover crop effect, the overall composition of metatranscriptionally active MAGs did not change by cover crop treatment, but rather time **(Additional File 9,** Fig. 6 & **Tables 4–5).** Yet we did observe that the active genera changed over the course of the experiment. For example, genera in the family *Nitrososphaeraceae* (phylum *Thermoproteota,* previously *Thaumarchaeota)* contributed roughly 50% of exudate-addition phase metatranscriptome expression (Fig. 4A, **green shading).** But by day 21 of enrichment, control and cover crop microcosm metatranscriptomes were dominated by MAGs from the *Bacteroidota,* including unclassified genera in the *Crocinitomicaceae* (genus *M2408)* and *JADKCL01* (genus *JADKCL01)* families (Fig. 4A). Comparing these membership and metabolite findings, the chemical data showed much stronger restructuring by treatment and time than the genus level active members.

To better identify specific organismal responses to individual cover crop treatments we next focused on genome (not genus) resolved responses within the microbial community. During exudate amendment, only a single MAG from an undescribed species in the *Actinomycetota* increased in activity in response to hairy vetch amendment *(JAJTCL01 sp021323255),* while a single MAG from a novel genus in the *Patescibacteria* (family *UBA1547)* was enriched in response to sorghum exudates **(Additional File 8).** Next, we identified MAGs that broadly responded to the cover crop exudates compared to the control. We found 23 MAGs were enriched in the control metatranscriptomes and 9 MAGs were enriched across the cover crop exudate treatment metatranscriptomes **(Additional File 9,** Fig. 7). The latter group included three MAGs in the *Nitrospiraceae* (including two in the *Nitrospira_C),* two MAGs in the *Gammaproteobacterial* genus *Hydrogenophaga,* one MAG classified as *Nitrobacter vulgaris,* and MAGs representing novel species of *Arthrobacter, Lacipirellula,* and *Paucimonas*. Collectively, this suggests cover crops produced root exudate compounds that stimulated specific microorganisms in the broader community.

Next, the impact of exudate addition on gene expression was evaluated by aggregating genes at the functional level (by annotation protein type). We identified 114 microbial functions that were enriched in the control metatranscriptome, and 145 functions that were enriched in the cover crop exudate amended microcosms, at day 5 **(Additional File 9, Fig. 8).** We observed distinct nitrogen cycling gene expression between control and exudate metatranscriptomes at day 5 (Fig. 4B; **Additional File 9, Figs. 8 &9).** Functions associated with nitrogen transport, mineralization and assimilation were discriminant to the control microcosm metatranscriptomes. In support of the microorganisms in the control microcosms being nitrogen limited, we confirmed the exudate metabolites contained organic nitrogen in the form of urea and several amino and organic acids while the controls received only water amendments **(Additional File 2).** Additionally, ammonia oxidation gene expression (*amoB*) was more enriched in the control, with contributions from four genera of *Thaumarchaeota* **(Additional File 8).** Nitrite reduction (*nirK)* was also enriched in the control, with an average of 97% control gene expression from the four *Thaumarchaeota* genera. The increased expression of *amoB* further supports the idea that the control soil microcosms are nitrogen limited, as genera from archaeal ammonia oxidizers were detected and active in all cover crop and control treatments (Fig. 4A), but this increased gene expression in controls may be due to the low ammonia environment [56].

The only nitrogen function enriched in the exudate microcosms was nitrite oxidation *(nxrB),* or the second step of nitrification resulting in nitrite being oxidized to nitrate. Gene expression was derived from *Nitrospira_C* MAGs and a MAG from an undescribed genus *WHTF01* in the *Binatia* **(Additional File 8).** Concomitant with this gene result, as mentioned above, a third of the discriminant cover crop transcriptionally active MAGs were bacterial nitrite oxidizers (*Nitrospira_C*, *Nitrobacter)*. The enrichment of bacterial nitrite oxidizers was unique to the cover crop treatments, suggesting these nitrifiers selectively responded to exudate components. Despite similar redox conditions between exudate and control reactors, in the control soils gene expression data suggest that nitrite is not oxidized but reduced in a closed loop (Fig. 4B). Instead in the controls, nitrite reduction was mediated by *Thaumarchaeota (nirK)* possibly to detoxify ammonification produced nitrite due to the absence of active nitrite oxidizers or was assimilated to ammonia (*nirBD)*. Finally, 8 genera contributed to *nirD* expression in the control, but 83% of the expression comes from 2 MAGs from the *Cellvibrio* and *Pseudomonas_E* genera. An additional 6 genera contribute to *nirB* expression in the control, with 45% of the expression from the same *Pseudomonas_E* MAG. These findings reflect an increase in control microorganisms to utilize specific nitrogen cycling functions due to the lack of available organic nitrogen (Fig. 4B). While we acknowledge limitations in translating these laboratory findings to the field scale, our multi-omics results do highlight how small-scale nutrient landscape changes caused by exudation could alter microbially expressed metabolic regimes (e.g., nitrification versus nitrite reduction) to result in disparate ecosystem manifested outcomes.

### Metabolite Evidence for Cover Crop and Microbially Produced Phytohormones

Given the recognized importance of microbial phytohormones in plant growth promotion, we next considered whether cover crop exudation could stimulate soil microbial phytohormone metabolism. We performed targeted metabolomics to quantify phytohormones in the microcosms, identifying 8 phytohormones that fluctuated over time: indole-3-acetic acid (IAA), indole-3-butyric acid (IBA), gibberellic acid 4 (GA_4_), 1-aminocyclopropane carboxylic acid (ACC), salicylic acid (SA), methyl salicylate (mSA), benzoic acid (BA), jasmonic acid (JA) (Fig. 5). Of these phytohormones, IAA, IBA, BA, GA_4_, and ACC had the strongest effect across the treatments (Fig. 5A; **Additional File 9, Tables 6–9).** Notably, only IAA and ACC were detected in cover crop exudate additions and were thus not detected in the water controls (Figs. 5B–C). Of the other phytohormones, IBA and GA_4_ were stimulated to be produced by the microbial community in response to added root exudates (Figs. 5D–E), while BA and mSA production was unique to the non-exudate stimulated controls **(Additional File 9, Fig. 10).** Our findings show that cover crop roots exude unique phytohormone profiles, but also that the soil microbiome likely encodes diversity of phytohormone producing and consuming metabolisms.

Of the different phytohormones we were particularly interested in ACC, IAA, IBA, and GA_4_ dynamics because they had clear cover crop signal. Our exudate metabolomics data confirmed ACC was detected as a component of the endogenous root exudate across all cover crops. As a result, ACC increased during the exudate addition phase (up to day 5). After day 7, ACC abundance stabilized (e.g., hairy vetch) or decreased (e.g., rapeseed, sorghum, cereal rye), the later indicating a possible microbial consumption (Fig. 5C). Like ACC, IAA was a product of cover crop direct addition in the hairy vetch but was not a component of other exudate treatments. However, the IAA accumulation signal was not as clear as ACC during the exudate addition phase (up to day 5) (Fig. 5B). After exudate stimulation stopped, IAA generally decreased in 3 cover crop treatments (hairy vetch, cereal rye, rapeseed), except for sorghum where IAA increased overtime (Fig. 5B; **Additional File 9, Table 10).** Similarly, IBA, an indole related to IAA, accumulated significantly only in the cereal rye at the post exudation phase (Fig. 5E; **Additional File 9, Table 6).** These latter two findings suggest the exudate chemistry stimulates members of the microbiome for indole production.

Based on these interesting indole cover crop responses, we mined our untargeted LC-MS data for additional indoles, identifying 2 unknown compounds that shared structural similarity to IAA (see Methods). Unknown indoles were from the chemical subclasses (i) indoles and (ii) indolyl carboxylic acids and derivatives and could be further categorized as a 3-alkylindole compound and an IAA derivative, respectively **(Additional File 9, Table 11).** The unknown IAA derivative was significantly more abundant in the rapeseed metabolomes compared to the control at each timepoint following day 1 **(Additional File 9, Fig. 11A & Table 12).** Exhibiting a broader response, the unknown 3-alkylindole was significantly enriched at each timepoint following the first day of exudate addition in rapeseed, sorghum, and hairy vetch compared to the control **(Additional File 9, Fig. 11B & Table 13).**

While there are more than 100 gibberellic acids (GA) known, only four (GA_1_, GA_3_, GA_4_, and GA_7_) have characterized biological activity [57]. Of these, GA_4_, a C20-gibberellin, was detected exclusively, and increasingly over time, in the cereal rye treated microcosms (Fig. 5D; **Additional File 9,Table 7).** Further supporting broader microbial GA production, we found two GA features that shared structural similarity with GA_4_ that were further characterized as type C20-and C19-GA **(Additional File 9, Table 11).** Given that GA compounds were not detected in the exudates, and they collectively increased after exudate stimulation in the cereal rye **(Additional File 9, Fig. 12A-B & Tables 14–15),** it is likely that cereal rye exudates contained compounds that stimulated microbial production of these GA. These findings show the power of combining untargeted metabolomics with targeted phytohormone quantification to enable tractability of microbial phytohormone biosynthesis. Our metabolite analyses highlight how much yet to be discovered biochemistry is likely residing in agricultural soils.

### Metatranscriptome Evidence for a Microbial Role in Phytohormone Transformations

Based on the possible ACC microbial consumption observed in our metabolite dynamics (Fig. 5C) and the critical role this compound plays as a recruitment cue for plant growth promoting bacteria [58], we mined ARM MAGs for the ACC deaminase (*acdS)* gene. Many bacteria use *acdS* to metabolize ACC via deamination, curbing the abiotic stress induced ethylene production and its adverse effect on plants [58]. In total, 66 genes from 63 MAGs were annotated as *acdS* **(Additional File 3).** As ACC deaminase genes are commonly misannotated as D-cysteine sulfhydrase [45, 46], we curated these putative hits for key active site residues to identify 13 high confidence *acdS* genes from distinct MAGs in the *Actinomycetota* (n = 9) and the *Pseudomonadota* (formerly *Proteobacteriota,* n = 4) (Fig. 6A).

Two of these *Actinomycetota* MAGs *(Pseudonocardia, Ornithinibacter)* expressed ACC deaminase across treatments and timepoints. These strains represent the most likely biological culprits contributing to ACC removal in the treatment microcosms. Analysis of the broader gene expression of these two MAGs suggests the α-ketobutyric acid generated from ACC deaminase activity could be used to support isoleucine synthesis for these microorganisms (Fig. 6B; **Additional File 3).** Additionally, the *Ornithinibacter* MAG expressed genes for participation in arabinan utilization, a plant polysaccharide found in high concentrations in roots [59], as well as the genes for nitrate reduction *(Nap)*. These paired metabolite and genome-resolved expression data uncover how cover crop ACC exudation can enrich for microorganisms with the capacity to modulate soil carbon and nitrogen cycles that benefit plant health.

Microbial biosynthesis of indole derivatives, like auxins, can impact plant health directing proper plant growth and development [60, 61]. Given that IAA was only a component of the hairy vetch exudate pool, the IAA detected in sorghum, cereal rye, and rapeseed was likely microbially generated. This compound and other closely related indole intermediates can be synthesized via five microbial pathways derived from tryptophan (Trp), all converging on the final oxidation step converting indole acetaldehyde (IAAId) to IAA by aldehyde dehydrogenase (*ALDH)* [61, 62]. We profiled genes for IAA pathways in the ARM database and found 4 pathways were represented in our MAGs (Fig. 7A, **Additional File 3).**

Of the 4 pathways, the indole-3-pyruvate (IPA) pathway was likely not contributing to the metabolite signal. We found 10 MAGs carrying IPA decarboxylase, yet we found no expression of either genes in the pathway (Fig. 6A). For the second pathway, the indole-3-acetamide (IAM) pathway, we failed to identify any MAGs that encoded both genes in this pathway. However, it has been suggested that this could be community metabolism [63], thus we still examined the gene expression patterns in our data. One MAG from the unnamed genera belonging to the *Geminicoccaceae* family expressed *iaaM* and a different MAG from the *Burkholderiales* order expressed *iaaH*. Expression of these two genes was highest in the rapeseed, hairy vetch, and cereal rye exudate treatments at day 5 (Fig. 7B). Our combined metabolite and metatranscriptome data suggest that this pathway was likely contributed to the IAA-like metabolite signal in the cereal rye and rapeseed.

The third pathway, the tryptamine (TAM) pathway, best explains the sorghum metabolite IAA production in later time points (Fig. 7B). This pathway is composed of two genes that were encoded across 116 MAGs, but 1 MAG from the *Woeseiaceae* family in the *Pseudomonadota* expressed both genes in the sorghum microcosms making this organism the most likely source for the increased IAA detected in sorghum metabolites at later time points. The fourth pathway, the indole-3-acetonitrile (IAN), is identified by the nitrilase gene. This gene was encoded by 55 MAGs, with expression detected in 5 MAGs from three genera in *Actinomycetota,* one in *Pseudomonadota,* and one in *Chloroflexota*. Expression was greatest in rapeseed at timepoints 5 and 21 (Fig. 7B). Holistically, our multi-omics indicates higher functional diversity expressed in the rapeseed treatment, where IAA synthesis genes were expressed by diverse organisms using two different pathways (IAN, IAM). Lastly, 66% of the MAGs in ARM had the potential for catalyzing the final step in IAA synthesis, 15 of which expressed this gene only in exudate microcosms during (day 5) or after exudate stimulation (day 21; Fig. 7B). Our findings clearly validate a role for cover crops in producing a chemical environment that promotes microbial IAA production.

Gibberellic acids (GA) are important phytohormones that promote plant root and stem elongation [64]. Because the cereal rye metabolites showed a clear microbial production signal, we next wanted to identify microbial taxa with this capacity. We found 176 MAGs that encoded at least one gene in the GA operon **(Additional File 9, Fig. 12C),** with 63 encoding *CYP115,* the gene responsible for converting GA_9_ into bioactive GA_4_ (Fig. 7C–D; **Additional File 3).** Supporting our GA_4_ metabolite data, there were 5 MAGs that expressed *CYP115* only in cereal rye metatranscriptomes at days 5 and 21 (Fig. 6A and E). A novel MAG in the *Thermomicrobiales* order *(Chloroflexota)* encoded 6 of 9 GA operon genes and actively expressed 5 (*CYP112, CYP114, CYP115, GGPS, SDR*) of the transcripts at day 21 in the cereal rye treatment and not in the control making this MAG the most likely culprit for GA_4_ production (Fig. 7D). *CYP115* expression by this MAG increased over time showing a significant positive correlation with GA_4_ metabolite production (Fig. 7F; **Additional File 9, Table 16).** Our findings showing *Thermomicrobiales* order may be important in the production of GA_4_, demonstrated the power of these laboratory experiments for uncovering new microbial physiologies with relevance to plant growth promotion.

## Discussion

### Cover Crop Root Exudate Stimulation Expands the Microbial Genomic Cataloging of the Agricultural Soil Microbiome

Cultivation-independent, community-level genomic techniques are increasingly necessary to untangle the mechanistic relationship between soil microbial community metabolism and plant root exudates. However, obtaining genome-resolved metagenomic data from agricultural field soils is challenging due to microorganismal richness and high strain diversity, the latter especially confounds existing metagenomic recovery methods [65]. Additionally, the physical and chemical heterogeneity that changes across millimeter scales, also confounds the linkages of plant chemical inputs to microbial responses. Furthermore, the complexity of soil management systems (e.g., tillage and fertilization practices) that impact soil microbial function also obscures the effects of cover crop-microbiome interactions in field scale studies. Thus, using intermediate complexity laboratory-scale ecosystems (microcosms) allows for the temporal resolution of microbial dynamics and linkages of microbial gene expression patterns to changes in soil chemistry.

These reactors were designed to illuminate the immediate impact of cover crop root exudation on agricultural soil microbiomes. As a result, this experimental design does not capture the myriad of ways cover crops can shape soil microbiomes (e.g., through root architecture and plant litter decomposition), nor were these batch-operated experiments conducive for tracking the legacy effect (months, years) of cover crops on soil microbial communities. While not a perfect replica of the complexity of cover cropping systems in a field environment, this research highlights how microcosms provided a controlled and replicable environment to construct a metabolic blueprint of agriculturally relevant taxa, many of which have not been genome sequenced or cultivated. Our approach and results provide mechanistic linkages between exudates and soil microbial communities that can inform future research questions and the design of field-scale experiments.

Here, we establish ARM, the first microbial genome catalog of plant root exudate-responsive agricultural soil community members. Agricultural soils were used as an inoculum and stimulated over 6 days of various exudate additions with the metabolite and microbial community responses tracked for 15 days post-stimulation in two different experiments that collectively make up ARM (Fig. 1). Coupled to ARM is an extensive catalog of metabolomics data from two cover crop exudate experiments, enabling us to decode the microbial membership, metabolic, and expression responses to exudate amendments from commonly used cover crops. Apart from providing multi-omic resources for ecologically relevant taxa, ARM MAGs offer access to genomic information from novel taxa as 70% of MAGs in the database lack a species assignment, and 87% are only recognized by alphanumeric identifier (Fig. 2). Our intention was to establish a publicly accessible repository to support fellow researchers engaged in agricultural soil microbiome studies. This repository aims to serve as a practical resource for enhancing the retrieval of microbial genomes in agricultural fields, promoting emerging knowledge of relevant taxa, specifically those involved in microbial phytohormone biosynthesis.

This experiment highlighted new expressed capabilities of soil microbiomes. For example, in agricultural soils, *Thaumarcheota* are well known ammonia oxidizers that govern fate of soil nitrogen. Here, we contribute to growing genomic catalog of these ecologically relevant taxa [66, 67], adding 11 total MAGs that belong to 6 genera, 5 of which were only identified by alphanumeric identifiers. Our metatranscriptome data shows these microbes are active in exudate-treated and unstimulated soils, especially during the initial phases of the experiment, thus providing insights that can guide further cultivation efforts. Interestingly, however, the second phase of ammonia oxidation, nitrite oxidation, was clearly enriched only in exudate reactors by non-comammox members of the *Nitrospira_C* genus, resulting in different nitrogen outcomes with and without exudation (Fig. 4). This finding highlighted how small chemical changes produced by cover crop exudation could result in differential ecosystem outputs, as others have shown [68].

Additionally, our phytohormone expression data revealed new genera that could be plant growth promoting taxa. For example, our data showed that members of currently undefined genera in the *Saprospiraceae (Bacteroidota),* the *GWC2-71-9 (Gemmatimonadota*), *Geminicoccaceae* and *Woeseiaceae (Pseudomonadota*) expressed genes for IAA or GA_4_ production (Fig. 6). Only two exudate-discriminant MAGs expressed the capacity for producing multiple phytohormones, one of these was the novel *Woeseiaceae*. Together our gene expression data show how cover crops through exudation can stimulate changes in nutrient cycling and phytohormone production.

### Root Exudate Amendments Alter Microbial Community Function and Metabolisms

Plant root exudates are known to shape the rhizosphere microbiome, an area of extraordinary microbial activity surrounding plant roots where abundant nutrients stimulate diverse organisms to colonize the rhizosphere. This hotspot of biological activity has the potential to influence plant and soil functional outcomes through the manipulation of root exudates. However, the intentional manipulation of the rhizosphere microbiome is limited in practice and has historically involved bacterial or fungal soil inoculation, such as inoculating seeds with PGP microorganisms, to encourage proliferation without the need for external chemicals [69–71]. The primary pitfall to inoculant-focused strategies is the lack of inoculant resilience to competition or lack of proliferation due to unfavorable soil conditions which decreases their impact. Alternatively, successful experiments using the complementary approach of altering root exudate chemistry to induce changes in the microbiome are sparse but suggest great potential [72]. Thus, understanding how variability in root exudation across plant species and genotypes can impact plant-microbial associations will provide important insights for exudate-focused intentional microbiome manipulation to enhance primary crop performance and sustainability. Here, our combined metabolite and metatranscript data from cover crop exudate and non-exudate microcosms revealed important microbial roles in the degradation of ACC and the production of IAA and GA_4_.

The phytohormone ACC is a precursor to the synthesis of ethylene in plants and has been shown to enhance stress resistance and relieve ethylene inhibition of plant growth. Our metabolite and metatranscript data suggested ACC consumption was possible in all exudate treated reactors. ARM contained 13 MAGs from the phyla *Actinomycetota* (n = 9) and *Pseudomonadota* (n = 4) that encoded the *acdS* gene (Fig. 6A), deaminating ACC into α-ketobutyric acid and ammonia (Fig. 6B). Again, highlighting metabolic novelty uncovered in these reactors, for all 9 of the *Actinomycetota* we could not find prior accounts for ACC deamination. This has important ramifications for designing microbiome informed cover cropping strategies, as there is growing awareness that ACC not only acts in ethylene regulation, but also attracts ACC-degrading bacteria to establish a positive rhizosphere interaction [73–76]. In fact, this gene has been cited as an important trait for plant growth promoting bacteria [58].

The important endogenous plant auxin, IAA, is necessary for directing proper plant growth and development with production capacity being widespread among plant-associated microorganisms [60]. Within ARM, we detected genomic evidence for four of the five known Trp dependent IAA biosynthetic pathways [61]. We found 215 MAGs encoded for at least one gene in any IAA pathway and 29 MAGs expressed one gene within any IAA pathway. Further, previous studies have demonstrated that bacteria are primed to produce IAA via the IPA or IAM pathways [61, 77], but our results highlight that the genomes analyzed here more dominantly encoded and expressed genes from the TAM and IAN pathways **(Additional File 9, Fig. 11C-D).** Out of the 15 MAGs expressing IAA synthesis, 20% and 33% expressed *DDC* and *MAO,* respectively (TAM pathway), while only 7% expressed *iaaM* and *iaaH* (IAM pathway), and 33% expressed nitrilase (IAN pathway) **(Additional File 9, Fig. 11C).** Our results suggest that MAGs encoding the IPA or IAM pathway were primarily from the *Pseudomonadota, Acidobacteroita,* or *Bacillota* phyla while MAGs encoding the TAM or IAN were much more diverse **(Additional File 9, Fig. 11D).** Our findings show that IAA production in soils is functionally redundant, and that multiple cover crop exudation strategies can upregulate indole biosynthetic routes. Future research to identify the exudate compounds that directly stimulate microbial phytohormone production could lead to new targets for cover crop breeding and their integration into biological precision agriculture approaches.

The GA phytohormones act endogenously in plants to promote seed germination, seedling growth, flowering and leaf expansion and influence plant-microbe interactions [78, 79]. Plant-associated bacteria can produce GA to stimulate primary root elongation and lateral root extension [80–82]. Here, we found cereal rye exudate amendments increased the microbial production of bioactive GA_4_ over the timeseries, with two other GA intermediates produced during the exudate-addition phase (Fig. 4E; **Additional File 9, Figs. 12A-B).** In terms of GA genetic potential, the core GA biosynthetic gene operon contains 8 genes and is widely distributed in rhizobia **(Additional File 9, Fig. 12C),** whereas *CYP115* has only been found in certain species and is required for GA_4_ synthesis [49, 83]. While 5 MAGs encoded and expressed *CYP115* exclusive to the cereal rye treatment, the MAG most likely responsible GA_4_ production in cereal rye was a member from the *Thermomicrobiales* order (*Chlroflexoata*). There are limited reports about the *Thermomicrobiales* in agricultural soils, yet 16S rRNA gene surveys have suggested members of this phylum are enriched in arid agricultural soils much like the soil inoculum used here [84]. Our findings uncovered bacteria that could be explored as plant growth promoting targets for enhancing bioactive GA_4_ production, potentially creating a favorable legacy environment for cash crops following a period of cereal rye cover cropping.

## Conclusions

Integrating a simplified and manageable laboratory soil system with advanced high-resolution multi-omics techniques afforded new insights into the intricate interplay between plant root exudates, the soil microbiome, and the capacity for soil microbial communities to contribute to phytohormone pools. We first provide ARM, a public agricultural genome database, creating a community resource for use in related microbiome-cover crop workflows that capture over 300 microbial genomes. Of these genomes, 215 encode hallmark genes of phytohormone metabolism, advancing the knowledge of the diversity of the physiology. Using ARM to contextualize our metabolite and metatranscriptome data, we then demonstrate cover crop exudate amendments applied at a physiologically relevant concentration impact microbial metabolism and membership function. Future studies can scale these hypotheses to the field to analyze the impact of plant genotype, root exudates, and associated microbial communities on overall plant performance. Additional research can detangle the precise chemical cues in these exudate cocktails that elicit direct microbial metabolic outcomes beneficial for soil and plant health. In summary, the results of this study represent an important step toward decoding the complex chemical language between plants and their rhizosphere microbial communities, a translation required to optimize these interactions for enhancing the environmental sustainability and productivity of agricultural systems.

## Data Availability

The datasets supporting the conclusions of this article are publicly available. Untargeted LC-MS/MS HILIC data can be found at the MassIVE data repository under the identifier MSV000092960 and final normalized data can be found in **Additional File 2.** Targeted phytohormone UHPLC-MS/MS quantification can be found in the **Additional File 2.** Metagenome shotgun sequencing data are available in NCBI under BioProject accession no. PRJNA725542 (for biosample accession numbers, see **Additional File 3).** The raw annotations for each ARM MAG are deposited on Zenodo, see **Additional File 6** for Zenodo DIO. Metatranscriptomics sequencing data are available for download at the JGI Joint Genome Portal under Project ID 142520. See **Additional File 7** for NCBI BioSample accession numbers and geTMM normalized values. All root exudate data generated and used as root exudate treatments in this study are included in **Additional File 2** and in the published manuscript by Seitz, et. al 2023. All scripts used in data processing are available at ThePrenniLab GitHub page under repository “Seitz_Microbiome_2024” (https://github.com/ThePrenniLab/Seitz_Microbiome_2024).

## Abbreviations

ARM: Agricultural Exudate-Responsive Metagenomic
MAG: metagenome-assembled genome
DI: deionized
MS: Murashige and Skoog
UHPLC-MS/MS: Ultra High Performance Liquid Chromatography-Mass Spectrometry
LC-MS/MS: Liquid Chromatography-Mass Spectrometry
SRM: Selected Reaction Monitoring
GTDB-tk: Genome Taxonomy Database ToolKit
DRAM: Distilled and Refined Annotation of Metabolism
ACC: 1-Aminocyclopropane carboxylic acid
acdS: 1-aminocyclopropane carboxylic acid synthase
GA_4_: gibberellic acid 4
CYP: cytochrome P450
PLS-DA: Partial Least Squares Discriminate Analysis
VIP: Variable Importance in Projection
NMDS: non-metric multidimensional scaling
geTMM: gene length corrected trimmed mean of M-values
amoB: ammonia oxidation
nirK: nitrite reduction
nxrB: nitrite oxidation
nirBD: nitrite reductase
IAA: indole-3-acetic acid
IBA: indole-3-butyric acid
SA: salicylic acid
mSA: methyl salicylate
BA: benzoic acid
JA: jasmonic acid
nap: nitrate reduction
IPA: indole-3-pyruvate
IAM: indole-3-acetamide
TAM: tryptamine
IAN: indole-3-acetonitrile
IAAId: indole acetaldehyde
Trp: tryptophan
ALDH: aldehyde dehydrogenase
iaaM: tryptophan 2-monooxygenase
iaaH: indoleacetamide hydrolase
DDC: aromatic-L-amino-acid/L-tryptophan decarboxylase
MAO: monoamine oxidase
ipdC: indole-pyruvate decarboxylase
GGPS: geranylgeranyl diphosphate
SDR: short-chain dehydrogenase/reductase
PGP: plant growth promoting
